# Heart rate recovery and blood pressure response during exercise testing in patients with microvascular angina

**DOI:** 10.1186/s40885-019-0108-x

**Published:** 2019-03-01

**Authors:** Bong-Joon Kim, Eun-Ah Jo, Sung-Il Im, Hyun-Su Kim, Jung Ho Heo, Kyoung-Im Cho

**Affiliations:** 1 Division of Cardiology, Department of Internal Medicine, Kosin University College of Medicine, 34, Amnam-dong, Seo-gu, Busan, 602-702 Korea; 2 Convergence Medicine & Exercise Science Research Institute, Kosin University College of Medicine, 34, Amnam-dong, Seo-gu, Busan, 602-702 Korea

**Keywords:** Microvascular angina, Cardiac autonomic function, Heart rate recovery

## Abstract

**Background:**

Angina pectoris with a normal coronary angiogram, termed microvascular angina (MVA), is an important clinical entity; however, its causes remain unclear. Autonomic dysfunction is one of the possible causes. Therefore, this study aimed to investigate parasympathetic dysfunction assessed by heart rate recovery (HRR) and increased sympathetic activity assessed by exaggerated blood pressure (BP) response (EBPR) to exercise in MVA.

**Methods:**

The study participants were consecutive patients with anginal chest pain who underwent both coronary angiography with an ergonovine provocation test and a treadmill exercise test between January 2008 and February 2015. Patients with significant coronary artery disease (coronary artery stenosis ≥50%) or significant coronary artery spasm (≥90%) were excluded. Based on the treadmill exercise test, patients were categorized into the microvascular angina (MVA) group (patients with uniform ST depression ≥1 mm) and the control group. HRR was defined as peak heart rate minus heart rate after a 1 min recovery; blunted HRR was defined as ≤12 beats/min. EBPR was defined as a peak exercise systolic BP ≥210 mmHg in men and ≥ 190 mmHg in women. These parameters were compared between patients with MVA and the controls.

**Results:**

Among the 970 enrolled patients (mean age 53.1 years; female 59.0%), 191 (20.0%) were diagnosed with MVA. In baseline characteristics, the MVA group had older participants, female predominance, and a higher prevalence of hypertension. The MVA group showed significantly lower HRR 1 min (24.9 ± 15.9 vs. 31.3 ± 22.7, *p* <  0.001) compared with the control group. Moreover, the proportion of EBPR was significantly higher in the MVA group than in the control group (21.5% vs. 11.6%, p <  0.001). Multivariable logistic regression analysis showed that age (odds ratio (OR), 1.045; 95% confidence interval (CI), 1.026–1.064; p <  0.001), HRR 1 min (OR, 0.990; 95% CI, 0.982–0.999; *p* = 0.022), and EBPR (OR, 1.657; 95% CI, 1.074–2.554; p = 0.022) were independently associated with MVA.

**Conclusion:**

HRR and EBPR were associated with MVA, which suggests a link between MVA and autonomic dysregulation.

## Introduction

Microvascular angina (MVA) is a clinical condition characterized by exertional angina, exercise-induced myocardial ischemia, and a normal coronary angiogram [1]. Despite the absence of significant coronary stenosis, MVA shows evidence of myocardial ischemia on stress tests [2]. In addition, quality of life in patients with MVA is often impaired by long-term, recurrent chest pain [3], and MVA may affect left ventricular systolic and diastolic function [4]. The precise mechanism remains unclear, and microvascular dysfunction, autonomic dysregulation, generalized vascular disorder, and abnormal subendocardial perfusion have been suggested [5]. Among them, autonomic dysfunction is one of the major possible causes of vasomotion in MVA [6].

An exercise treadmill test, easy to apply in clinical practice, can detect the presence of myocardial ischemia and changes in heart rate and blood pressure (BP) during exercise. Heart rate recovery (HRR) is a simple noninvasive measurement related to autonomic nervous system dysfunction that indicates impaired parasympathetic reactivation [7–9]. Previous studies have shown that blunted HRR, defined as a ≤ 12 beats/min decrease in heart rate (HR) from peak exercise to 1 min into recovery, is a powerful predictor of overall mortality [7, 10].

Blood pressure response during graded exercise is a useful parameter to predict hypertension, [11] and is associated with adverse cardiovascular outcomes [12, 13]. In particular, exaggerated BP response (EBPR) to exercise is related to impaired endothelial vasodilator function [14] and is also thought to be a predictable marker of masked hypertension (MHT) [15].

Therefore, this study aimed to investigate autonomic dysfunction as assessed by HRR and increased sympathetic activity as assessed by EBPR in MVA.

## Methods

This is cross-sectional, observational, single-center cohort study. We retrospectively reviewed a total of 1393 patients without significant coronary artery disease (coronary artery stenosis ≥50%) who underwent both CAG with ergonovin provocation test and treadmill test during between January 2007 and August 2015. We excluded the 300 patients with significant coronary spasm (≥90%) on ergonovin provocation test and 29 patients with other exclusion criteria as follows; any systemic disease such as significant liver disease, neurologic disorder, or malignant disease; secondary hypertension; history of heart failure; valvular heart disease; atrial fibrillation. In enrolled 998 patients, 28 patients were excluded due to outlier value in statistical analysis. MVA was defined as follows: (1) typical stable angina induced by effort, (2) a flat or downsloping depression of the ST segment > 0.1 mV below baseline and lasting longer than 0.08 s during the treadmill test, and (3) normal or near normal coronary arteries on coronary angiogram (vascular wall irregularities or discrete very mild stenosis (< 20%)) [16]. Demographic characteristics recorded at the first visit comprised age, sex, height, weight, current medications, smoking history, and other diseases. Blood samples were drawn for measurement of total serum cholesterol, triglycerides, high-density lipoprotein and low-density lipoprotein cholesterol, blood glucose, creatinine, uric acid, and high sensitivity C-reactive protein. Body mass index (BMI) was calculated as the ratio of dry weight in kilograms to height in square meters.

### Echocardiographic measurement

Standard 2-dimensional echocardiography was performed on all subjects lying in the left lateral decubitus position using a 3.5-MHz transducer (Philips iE33, Philips Medical Systems, Bothell, WA, USA), and the echocardiography examiners were blinded to patient information. Measurements of the diameter of the left ventricle (LV) cavity, the thickness of the interventricular septum and posterior wall, and the LV mass index (LVMI) were performed according to criteria outlined by the American Society of Echocardiography [17]. Pulsed wave Doppler of transmitral LV inflow was performed in the apical four-chamber view, with the sample volume placed at the level of the mitral valve tips; Doppler variables were analyzed during three consecutive beats. The following measurements of global LV diastolic function were determined: peak early (E) and late (A) diastolic mitral flow velocity, their ratio E/A, and early (Ea) diastolic mitral annular velocity.

### Exercise treadmill testing

All patients underwent symptom-limited exercise stress testing (GE CASE T2100; GE Medical Systems, Milwaukee, WI, USA) according to the protocol by Bruce et al. [18]. BP was measured using an automated BP monitor (Suntech Tango; Suntech Medical, Morrisville, NC, USA) throughout the treadmill test using the same arm as was used to measure resting BP. Twelve-lead electrocardiography was monitored continuously and printed at a paper speed of 25 mm/s; measurements of HR and BP were recorded at the end of each 3-min stage, at peak exercise, and at 1-min and 2-min intervals throughout recovery. The participants continued to exercise until volitional fatigue or if HR exceeded 95% of estimated maximal HR (220 bpm, age). The total exercise time was also recorded. Functional capacity was estimated in metabolic equivalents (METs) on the basis of the speed and grade of the treadmill [19]. During the recovery phase, the subjects continued to walk for 60 s at a speed of 1.5 mph, and then they sat down for 3 min with continued monitoring of BP, HR, and heart rhythm. HRR was defined as decrease in HR from peak exercise to 1 min and 2 min after cessation of exercise.

### Ergonovine test for provocation of coronary spasm

After diagnostic coronary angiography, incremental doses of ergonovine were injected intravenously (50, 100, 200 μg) over 10 s [20]. Two minutes after each injection, the coronary angiogram, electrocardiogram, BP, and patient symptoms were assessed. Coronary spasm was defined as near total or localized spasm (≥90% diameter) of the focal epicardial coronary artery with signs of chest pain or ischemic ST changes according to the Guidelines for Diagnosis and Treatment of Patients with Vasospastic Angina [21]. Intracoronary injection of isosorbide dinitrate was performed on completion of the ergonovine test, regardless of whether or not coronary spasm was confirmed.

### Statistical analysis

Statistical analyses were performed using the commercially available computer program SPSS 18.0 for Windows (IBM, Chicago, IL, USA). Data are presented as mean ± standard deviation for continuous variables and as percentages (%) if the data are categorical. The Mann–Whitney U test was used for continuous variables, and the Chi-square test was used for categorical data. The normality of data was tested using the Kolmogorov–Smirnov test. To identify independent contributors for MVA, univariate and multivariate logistic regression analyses were performed. A *p*-value < 0.05 was considered statistically significant.

## Results

A total of 970 patients were enrolled; the mean age was 53.1 years, and 59.0% were female. Among them, 191 were diagnosed with MVA, documented by no significant coronary stenosis with a positive exercise test, and 779 patients with a negative exercise test were compared as controls. In baseline characteristics, the MVA group was older, had a higher proportion of female participants, and had higher prevalence of hypertension and dyslipidemia. No significant differences were observed in BMI, office systolic/diastolic BP, and use of renin-angiotensin system blocker, beta blockers, calcium channel blockers, and statins (Table 1). Laboratory tests showed no significant differences between groups (Table 2). In echocardiogram parameters, the MVA group showed higher LV septal wall thickness, LV mass index, LA diameter, LA volume, and Ea compared with the control group (Table 3).

**Table 1:** Baseline characteristics

	Control (*n* = 779)	MVA (*n* = 191)	*p*-value
Age, years	52.0 ± 10.9	57.7 ± 9.1	< 0.001
Female gender, n (%)	440 (56.5)	132 (69.1)	0.001
Body mass index, kg/m^2^	23.3 ± 6.0	24.0 ± 6.6	0.221
Systolic BP, mmHg	127.1 ± 18.4	129.1 ± 16.6	0.262
Diastolic BP, mmHg	78.8 ± 12.6	78.0 ± 12.4	0.514
Smoking, n (%)	133 (25.5)	24 (17.9)	0.070
Alcohol, n (%)	179 (34.4)	30 (22.4)	0.009
Hypertension, n (%)	141 (26.7)	57 (37.3)	0.019
Diabetes mellitus, n (%)	80 (14.4)	27 (19.7)	0.146
Dyslipidemia, n (%)	212 (40.1)	69 (51.9)	0.018
Hyperthyroidism, n (%)	51 (9.6)	9 (6.8)	0.510
Medication			
Aspirin, n (%)	117 (15.0)	34 (17.8)	0.422
RAS blocker, n (%)	91 (11.7)	33 (17.3)	0.110
Beta blocker, n (%)	69 (8.9)	25 (13.1)	0.141
Calcium channel blocker, n (%)	97 (12.5)	31 (16.2)	0.314
Diuretics, n (%)	21 (2.7)	6 (3.1)	0.769
Nitrates, n (%)	79 (10.1)	17 (8.9)	0.871
Statin, n (%)	83 (10.7)	22 (11.5)	0.218

Data are presented as mean ± SD or frequency with percentage in parenthesis. *MVA* microvascular angina, *BP* blood pressure, *RAS* renin-angiotensin system

**Table 2:** Laboratory test

	Control (n = 779)	MVA (n = 191)	*p*-value
Hemoglobin, g/dL	13.5 ± 4.4	13.4 ± 2.5	0.695
White blood cells, 10^3^/μL	7.3 ± 3.7	7.1 ± 2.3	0.611
Platelets, 10^3^/μL	221.6 ± 56.1	216.8 ± 55.7	0.291
Uric acid, mg/L	5.4 ± 1.9	5.2 ± 1.5	0.374
Creatinine, mg/dL	0.8 ± 0.2	0.8 ± 0.8	0.083
Fasting glucose, mg/dL	100.1 ± 23.2	101.0 ± 24.2	0.674
Total cholesterol, mg/dL	175.7 ± 36.2	178.9 ± 38.0	0.315
LDL cholesterol, mg/dL	104.3 ± 48.6	104.0 ± 32.1	0.942
HDL cholesterol, mg/dL	47.9 ± 13.3	49.4 ± 16.2	0.221
Triglycerides, mg/dL	128.2 ± 119.8	119.0 ± 58.7	0.327
hs-CRP, mg/dL	0.3 ± 0.9	0.2 ± 1.0	0.384
TSH, uIU/mL	2.6 ± 7.4	2.2 ± 2.5	0.682
Free T4, ng/dL	1.5 ± 5.3	1.2 ± 0.7	0.649
T3, ng/dL	94.8 ± 30.8	91.7 ± 24.7	0.348

Data are presented as mean ± SD. *MVA* microvascular angina, *LDL* low density lipoprotein, *CRP* C-reactive protein, *TSH* thyroid stimulating hormone

**Table 3:** Parameters of Echocardiogram

	Control (*n* = 779)	MVA (*n* = 191)	*p*-value
LVEDD, mm	45.5 ± 4.6	46.0 ± 4.6	0.550
LVESD, mm	28.3 ± 4.3	28.0 ± 4.4	0.388
IVSTd, mm	11.2 ± 2.0	11.9 ± 2.3	0.001
PWTd, mm	10.0 ± 1.7	10.3 ± 1.6	0.100
LVMI, g/m^2^	112.2 ± 37.2	121.6 ± 40.9	0.011
LV EF, %	67.9 ± 7.7	69.4 ± 7.9	0.049
LA diameter, mm	34.0 ± 5.4	35.3 ± 5.7	0.013
LA volume, mL	16.5 ± 5.4	19.8 ± 7.3	< 0.001
E velocity, cm/sec	0.7 ± 0.2	0.7 ± 0.2	0.104
A velocity, cm/sec	0.6 ± 0.2	0.7 ± 0.2	< 0.001
Ea, cm/sec	0.08 ± 0.03	0.07 ± 0.02	0.005
E/Ea	9.1 ± 3.2	10.3 ± 3.3	< 0.001

All values are presented as the mean ± SD. *MVA* microvascular angina, *LVEDD* left ventricular end-diastolic diameter, *LVESD* left ventricular end-systolic diameter, *IVSTd* diastolic interventricular septal wall thickness, *PWTd* diastolic posterior wall thickness, *LVMI* left ventricular mass index, *RWT* relative wall thickness, *EF* ejection fraction, *LA* left atrial diameter, *E* peak early diastolic mitral filling velocity, *Ea* mitral septal annular velocity, *A* peak late diastolic mitral filling velocity

Table 4 summarizes the results of the exercise test. The MVA group showed significantly lower exercise time, METs, HRR 1 min (24.9 ± 15.9 vs 31.3 ± 22.7, *p* <  0.001), and HRR 2 min (64.1 ± 17.5 vs. 68.1 ± 15.5, *p* = 0.004) compared with the control group. Moreover, no differences were observed in resting systolic and diastolic BP between groups; however, the proportion of EBPR was significantly higher in the MVA group compared to the control group (21.5 vs. 11.6%, p <  0.001). Multivariable logistic regression analysis showed that age (odds ratio (OR), 1.045; 95% confidence interval (CI), 1.026–1.064; p <  0.001), HRR 1 min (OR, 0.990; 95% CI, 0.982–0.999; *p* = 0.022), and EBPR (OR, 1.657; 95% CI, 1.074–2.554; p = 0.022) were independently associated with MVA (Table 5).

**Table 4:** Parameters of Exercise test

	Control (*n* = 779)	MVA (*n* = 191)	*p*-value
Exercise time, min	7.9 ± 2.0	6.7 ± 2.4	< 0.001
Metabolic equivalents	9.8 ± 3.3	8.8 ± 5.2	0.001
Rest heart rate, bpm	69.5 ± 12.4	67.3 ± 11.6	0.028
Max heart rate, bpm	153.6 ± 18.6	144.7 ± 21.7	< 0.001
HR at recovery 1 min, bpm	122.3 ± 26.4	119.8 ± 25.7	0.238
HR at recovery 2 min, bpm	85.8 ± 15.1	81.0 ± 14.3	< 0.001
HRR 1 min, bpm	31.3 ± 22.7	24.9 ± 15.9	< 0.001
HRR 2 min, bpm	68.1 ± 15.5	64.1 ± 17.5	0.004
Blunted HRR, n (%)	236 (30.3)	53 (27.7)	0.537
Rest systolic BP, mmHg	126.5 ± 38.5	127.5 ± 14.2	0.731
Rest diastolic BP, mmHg	75.2 ± 10.8	74.5 ± 11.2	0.428
Max systolic BP, mmHg	171.4 ± 19.8	176.9 ± 23.9	0.004
Max diastolic BP, mmHg	81.1 ± 11.8	83.7 ± 11.7	0.007
EBPR, n (%)	90 (11.6)	41 (21.5)	< 0.001

Data are presented as mean ± SD or frequency with percentage in parenthesis. *MVA* microvascular angina, *HR* heart rate, *HRR* heart rate recovery, *BP* blood pressure, *EBPR* exaggerated blood pressure response

**Table 5:** Risk factors associated with the microvascular angina according to logistic regression models

	Univariable		Multivariable	
Risk factors	Odds ratio (95% CI)	*p*-value	Odds ratio (95% CI)	*p*-value
Age	1.058 (1.040 to 1.076)	< 0.001	1.045 (1.026 to 1.064)	< 0.001
Female gender	1.724 (1.229 to 2.417)	0.002	1.387 (0.964 to 1.995)	0.078
HRR 1 min	0.986 (0.978 to 0.993)	< 0.001	0.990 (0.982 to 0.999)	0.022
HRR 2 min	0.983 (0.972 to 0.994)	0.002	0.992 (0.980 to 1.003)	0.154
EBPR	2.093 (1.390 to 3.151)	< 0.001	1.657 (1.074 to 2.554)	0.022

*HRR* heart rate recovery, *EBPR* exaggerated blood pressure response

## Discussion

The major findings obtained from this study were as follows: (1) HRR for both 1 and 2 min was lower in patients with MVA, (2) the EBPR proportion was higher in the MVA group, and (3) HRR for 1 min and EBPR were independent predictors of MVA. These findings suggest a possible link between MVA and autonomic dysregulation.

Although the mechanism of MVA remains unclear and the terminology is confusing, studies have shown association with autonomic dysfunction. Lanza et al. demonstrated that metaiodobenzylguanidine (MIBI) uptake score is higher in patients with syndrome X, and impairment in cardiac MIBI uptake is associated with a reduction in HR variability indices [22]. Parasympathetic reactivation is an important factor in predicting autonomic dysfunction; as the principal determinant of the decrease in HR during early recovery, this mechanism is independent of age and exercise intensity [23]. Given the prognostic significance of diminished parasympathetic tone at rest, post-exercise HRR offers a noninvasive and feasible method to assess parasympathetic activation [23, 24]. Reduced HRR after exercise is a powerful predictor of overall mortality in patients without a history of heart failure or coronary revascularization [10]. Because it is simple to calculate from data obtained from standard exercise tests and does not require either 24-h Holter monitoring or specialized baroreflex sensitivity testing, HRR may be useful for assessment of risk in routine clinical practice.

Sympathetic activity is also an important factor of autonomic function, and BP response during exercise is an especially useful parameter. We observed that the MVA group had a higher prevalence of EBPR, suggesting that MVA might have impaired the vascular response. Vascular resistance is involved with multiple factors such as atherosclerosis, vessel spasms, and endothelial dysfunction. BP reflect the change in cardiac output during exercise or recovery can lead to changes in systolic BP [25]. Cardiovascular reactivity to both isometric and dynamic exercise has been shown to be one of the most important markers for predicting hypertension [11]. Impaired vascular function, including increased arterial stiffness and abnormal endothelial function, is associated with increased exercise BP response [12]. Among the parameters of BP response, exercise BP response is an important marker of cardiovascular risk that is associated with cardiovascular mortality [26, 27]. In particular, exaggerated exercise BP is a significant predictor for total cardiovascular events and for new onset of resting hypertension [27]. In our data, the MVA group had a higher prevalence of hypertension and higher LV septum thickness and LV mass index compared with the control group. Patients with hypertension or LVH may have frequent BP elevations due to underlying vascular dysfunction to compensate for increased cardiac output in low to moderate exercise [12]. So, we think these factors have affected our results somewhat.

Coronary spasm is one of the major components of chest pain in patients without coronary artery stenosis and it accounts for a large number of unexplained case. Vagal withdrawal is often a component of the mechanisms leading to spontaneous coronary vasospasm [28]. Decreased parasympathetic nervous activity with enhanced sympathetic nervous activity at night is reported to be involved in the mechanism underlying multi-vessel coronary spasm [29]. It is of note that we excluded patients with coronary artery spasm based on the ergonovine provocation test to rule out the effect of coronary spasm. Therefore, our findings demonstrated that, even after the effect of coronary spasm was excluded, autonomic dysfunction remained one of the important mechanisms of MVA.

## Limitations

This study has several important limitations. First, in baseline characteristics, the participants in the MVA group were older, more likely to be female, and had higher prevalence of hypertension and dyslipidemia. In particular, the higher prevalence of hypertension and trend toward higher use of anti-hypertensive medications may directly affect the BP response during exercise and change of HR. This is a major limitation of our study. Second, this was a retrospective study. Third, the definition of MVA was insufficient, and there was no evidence to suggest ischemia based on other imaging studies such as cardiac MRI or myocardial SPECT. Fourth, although we excluded patients with significant coronary spasm, we could not exclude the effect of microvascular spasms. Fifth, because this study was performed at a single tertiary care center, it is possible that biases existed with respect to patient referral and population sampling. However, we believe the results of this study show a valuable trend in MVA, especially regarding the use of HRR to evaluate this condition.

## Conclusion

Patients with MVA showed reduced HRR and greater EBPR compared with the control group, which suggest that there is a link between MVA and autonomic dysregulation.
